# Autonomic balance determines the severity of COVID-19 courses

**DOI:** 10.1186/s42234-020-00058-0

**Published:** 2020-11-24

**Authors:** M. Leitzke, D. Stefanovic, J.-J. Meyer, S. Schimpf, P. Schönknecht

**Affiliations:** 1 Department of Anesthesiology, Helios Clinics, Colditzer Straße 48, 04703 Leisnig, Germany; 2 Drägerwerk AG & Co. KGaA, Moislinger Allee 53–55, 23558 Lübeck, Germany; 3 Medical faculty of Leipzig University, Saxon Hospital, Hufelandstraße 15, Sächsisches Krankenhaus, 01477 Arnsdorf, Germany

**Keywords:** COVID-19, Cytokine storm, Cholinergic anti-inflammatory pathway, NF-κB pathway, Vagal nerve, Stimulation, Heart rate variability

## Abstract

COVID-19 has left mankind desperately seeking how to manage dramatically rising infection rates associated with severe disease progressions. COVID-19 courses range from mild symptoms up to multiple organ failure and death, triggered by excessively high serum cytokine levels (IL 1β, IL 6, TNF α, IL 8). The vagally driven cholinergic anti-inflammatory pathway (CAP) stops the action of nuclear factor κB (NF-κB), the transcriptional factor of pro-inflammatory cytokines. Thus, well-balanced cytokine release depends on adequate vagal signaling. Coronaviruses replicate using NF-κB transcriptional factor as well. By degrading the cytoplasmatic inhibitor of NF-κB subunits (IκB), coronaviruses induce unrestricted NF-κB expression accelerating both, virus replication and cytokine transcription.

We hypothesize that CAP detriment due to depressed vagal tone critically determines the severity of COVID-19.

## SARS-CoV-2 has set the world on fire

The severe acute respiratory syndrome corona virus 2 (SARS-CoV-2) shows broad genetic similarities with other coronaviruses (SARS-CoV, SARS-CoV-RaTG13) (Palayew et al. 2020). However, its affinity to cellular angiotensin converting enzyme 2 (ACE 2) receptors is 10-fold higher than that of SARS-CoV (Wang et al. 2020a). This explains the high contagiousness, which led to a pandemic within a few months after its appearance in Wuhan in the People’s Republic of China. The explosive spread of COVID-19, caused by SARS-CoV-2, forced the World Health Organization (WHO) to declare a Public Health Emergency of International Concern (PHEIC) on January 30th (Wang et al. 2020a). Moreover, the consequences of this major threat to health care systems and the accompanying economic and social lockdown in most of the countries are incalculable. This underlines the urgent need for therapeutic and preventive solutions to combat COVID-19.

In our clinical practice, the symptoms of SARS-CoV-2 infection presented along a wide range; from mild disease courses with influenza-like symptoms up to acute respiratory distress syndrome (ARDS) (see Glossary), and death. Despite ARDS we saw patients with liver damage, severe intestinal dysfunction, rhabdomyolysis, acute renal failure, and coagulopathy with subsequent hemorrhage or embolism. In the critically-ill patients, we saw excessively high pro-inflammatory cytokine serum levels (interleukin 1β - IL 1β (see Glossary), interleukin 6 – IL 6 (see Glossary), tumor necrosis factor α - TNF α (see Glossary), interleukin 8 – IL 8 (see Glossary)) and the extent of their hyperexpression was of high prognostic relevance. Concerning this, many authors speak about a hyperinflammatory syndrome or a cytokine storm (Mehta et al. 2020; Feldmann et al. 2020; Moore and June 2020).

Which course this potentially lethal infectious disease takes, is mainly dependent on where the overwhelming inflammatory cascade stops itself or can be therapeutically interrupted.

Elderly patients and patients with cardiovascular, respiratory, or metabolic disorders, as well as patients with cancer are most likely to have a severe course of COVID-19 (Jordan et al. 2020) (see Table 1). The question then arises: Which immunological commonality do these conditions share? And if there is commonality: Is this common immunological defect capable of causing those hyperinflammatory COVID-19 courses?

**Table 1:** Conditions posing a risk of severe COVID-19 courses

• Asthma	
• Chronic kidney disease being treated with dialysis	
• Chronic lung disease	
• Diabetes	
• Hemoglobin disorders	
• Immunocompromised	
• Liver disease	
• People aged 65 years and older	
• People in nursing homes or long-term facilities	
• Serious heart conditions	
• Severe obesity	
CDC/Centre for Disease Control and Prevention	
U.S. Department of Health & Human Services	

All these predisposing medical conditions have an imbalance of the autonomic nervous system (ANS) in common (Jarczok et al. 2019; Dalise et al. 2020). This is to the detriment of autonomic parasympathetic compartment and can be interpreted as an illness-adaptive sympathetic overexcitation to maintain homeostasis (Goldberger et al. 2019). But vagal signaling essentially controls cytokine expression and release by blocking nuclear factor κB (NF-κB) (see Table 2), the transcriptional factor (TF) of pro-inflammatory cytokines (Borovikova et al. 2000; Tracey 2007). Moreover, like cytokine transcription, the replication of coronavirus is promoted by NF-κB (Poppe et al. 2017). For its self-replication, the virus hijacks the NF-κB pathway (Poppe et al. 2017) neutralizing NF-κB subunit inhibitors. Under physiological conditions, this transcriptional pathway is restricted by vagal signaling. Thus, despite unrestricted virus replication, the virus-driven, uncontrolled acceleration of the NF-κB action leads to excessive cytokine transcription, inducing the cytokine storm (Sallenave and Guillot 2020).

**Table 2:** Transcriptional factor nf- κB – the key to pathology

**NF-κB** (**nuclear factor kappa-light-chainenhancer of activated B cells**) is a protein of complex structure that controls DNA transcription, cytokine production and cellular survival. NF-κB can be found in almost all animal cell types and promotes cellular reaction to stimuli such as stress, cytokines, free radicals, heavy metals, ultraviolet irradiation, and microbial antigens. NF-κB plays a principal role in regulating the immune response to infectious assault. Regulation disorders of NF-κB have been linked to viral infection, septic shock, hyperinflammatory and autoimmune diseases, cancer, and disturbed immune maturation. Finally, NF-κB has also been shown to be implicated in mechanisms of synaptic plasticity and memory.	

Because the pro-inflammatory NF-κB-pathway plays a pivotal role in several SARS-CoV-2 associated pathomechanisms and its control underlies mainly vagal signaling capacity, the modulation of impaired vagus nerve activity due to pre-existing conditions seems to be worth exploring further in COVID-19 patients.

## Cellular invasion of SARS-CoV-2, immune response and resulting tissue damage

### Cellular invasion

For coronaviruses two preconditions must be given to allow cellular invasion. First, the viral spike- or S-protein (see Glossary) must couple to the angiotensin converting enzyme 2 (ACE2) receptor at the surface of susceptible cells (Hoffmann et al. 2020). Second, the S-protein must be proteolytically primed by the transmembrane protease serine subtype 2 (TMPRSS2) beforehand (Hoffmann et al. 2020). TRMPRSS2 is mostly found on the epithelial cells in the respiratory tract, allowing cellular fusion between coronavirus and cell (Hoffmann et al. 2020). It cleaves the viral S-protein into S1 and S2 subunits. This cleavage enhances cellular adherence of the virus and promotes its endosome-independent cell entry. Due to this tissue tropism, SARS-CoV-2 yields high replication rates in the cells of the upper airways and pulmonary inflammation sites (Hui et al. 2020).

It was shown that especially epithelial cells of upper and lower airways such as monocytes, macrophages, and dendritic cells are capable of releasing cytokines along coronavirus infections (Dienz et al. 2012). SARS-CoV-2 RNA presents as a pathogen associated molecular pattern (PAMP) (see Table 3) in those cells and is recognized and captured by pattern recognition receptors (PRR’s) (see Glossary) (i.e. endosomal Toll-like receptors TLR3, TLR7, TLR8 and TLR9) inducing NF-κB activation with subsequent downstream release of NF-κB dependent cytokines (Poppe et al. 2017; Cervantes et al. 2012; Dosch et al. 2009; Moreno-Eutimio et al. 2020). Despite accelerating the inflammatory NF-κB pathway, PAMP recognition and damage-associated molecular patterns (DAMPs) (see Table 3) lead to inflammasome assembly. Inflammasomes are multimeric protein complexes composed of cytoplasmic sensors together with apoptosis-associated speck-like protein containing a caspase activation and recruitment domain (ASC or PYCARD) (see Glossary), and pro-caspase-1 (Lara et al. 2020). Receptor protein activation attracts ASC and caspase-1 to assemble an inflammasome (i.e. NLRP3)(see Glossary). This assembly induces self-cleavage and caspase-1 activation. Activated caspase-1 causes the release of IL 1β, IL 18 (see Glossary), and other inflammatory factors. Additionally, it leads to pyroptotic cell death, which promotes clearance of pathogens and damaged cells.

**Table 3:** **Molecular patterns of pathologic impact**

**Damage-associated molecular patterns** (**DAMPs**), are also described as danger associated molecular patterns, danger signals, or alarmin. They are nuclear or cytosolic biomolecules of host organisms that can initiate and perpetuate inflammatory response to *noninfectious* stimuli such as cellular damage in general. Following their release from the damaged cell or damage related cellular surface expression DAMPs move from a reducing to an oxidizing environment leading to protein denaturation.	
In contrast, **pathogen-associated molecular patterns (PAMPs)** evoke and perpetuate the inflammatory cellular response to *infectious* pathogens. Nucleic acid variants normally associated with viruses, such as doublestranded RNA (dsRNA), recognized by toll-like receptors (TLR3) as well as bacterial lipopolysaccharides (LPSs), endotoxins found on the cell membranes of gram-negative bacteria, are considered to be the prototypical class of PAMPs.	

IL1β is a principal mediator of systemic inflammation. It induces the expression of a large amount of pro-inflammatory genes and acts on numerous target organs (Jéru and Amselem 2011). Furthermore it is supposed to cause the additional release of pro-inflammatory cytokines involving IL 6, IL 8 and TNF α (Lucherini et al. 2018). Since IL 8 drives chemotactic migration of additional inflammatory cells, it promotes the perpetuation of the inflammatory process.

### Tissue damage and systemic cytokine distribution

The cytokines of the IL 1 family cause fever and inflammatory fibrotic conversion of pulmonary stroma. Histologic studies of pulmonary tissue from COVID-19 patients revealed diffuse alveolar damage due to infiltration of mononuclear cells and macrophages, as well as diffuse thickening of the alveolar wall forming a hyaline membrane due to virus related cytokine liberation (Xu et al. 2020). Despite perivascular T-cell infiltration, the vasculature of lungs from patients with COVID-19 also showed severe endothelial injury. This was associated with the presence of intracellular virus and disrupted cell membranes. Moreover, histologic analysis of pulmonary vessels in patients with COVID-19 revealed widespread thrombosis with microangiopathy (Ackermann et al. 2020). Taken together, the described mechanisms represent the dramatic pulmonary tissue damage with subsequent critical respiratory dysfunction that is seen in severe cases of COVID-19 (Conti 2020). Moreover, the self-enhancing cytokine pathways stand for the systemic flood of pro-inflammatory cytokines called cytokine storm (Mehta et al. 2020). At the cardiac level, cytokine storm related interleukins (IL 6, IL 1, TNFα) are known to be involved in inhibiting cardiomyocytic membrane channels (hERG-K+ channels/Ca + channels) (Lazzerini et al. 2020a) which take response for myocardial repolarization. Cytokine related impact on these channels is called inflammatory cardiac channelopathy (Lazzerini et al. 2020a). This in turn increases the risk of QT-prolongation related ventricular arrhythmias, significantly contributing to multi-organ dysfunction (Lazzerini et al. 2020a). The mechanisms of direct infection-related cardiomyocytic damage demonstrated by significantly elevated troponin-T levels remain speculative (Lazzerini et al. 2020a).

The cause of the dysfunctions in several other extrapulmonary organs consisting of tissues with few or no ACE 2 receptors (Fu et al. 2020) mentioned above remains unclear. It could be attributed to a direct virus attack or, more likely, a result of the locally or systemically impaired tissue oxygen supply due to infection associated coagulopathy which is in the majority of cases a coagulation predominant-type coagulopathy with embolism and ischemia resulting in tissue damage (Zuo et al. 2020).

## COVID-19 - autonomic imbalance, hyperinflammation, and viral replication

### The autonomic imbalance

With regard to the patient groups with an increased risk of a more severe progression of the disease, it can be seen that, all medical conditions predisposing for severe COVID-19 courses have a disturbed balance of the autonomic nervous system (ANS), with underrepresentation of the vagal branch in common (Jarczok et al. 2019; Dalise et al. 2020). This is due to the necessity that any impaired organ function must be compensated by additional performance from undisturbed organs to maintain homeostasis. This requirement is described as *extrinsic* autonomic dysfunction and is conducted via sympathetic overexcitation (Goldberger et al. 2019). In contrast to the *extrinsic* autonomic dysfunction, there is the *intrinsic* autonomic dysfunction describing a primary damage to the fibers of the autonomic nervous system. The leading cause for *intrinsic* autonomic dysfunction is diabetes mellitus (Goldberger et al. 2019). Histological evaluations in diabetic subjects have shown loss of and/or damage to myelinated vagus nerve axons. These findings provide evidence of the autonomic nervous system damage caused by diabetes, which is most likely a result of the hyperglycemia. (Duchen 1980). Thus, dispositional augmented sympathetic tone, and impaired parasympathetic autonomic signaling respectively, are part of all the disease patterns related to severe COVID-19 courses.

Despite the aforementioned conditions with concurrent autonomic balance disorders, several authors indicate that genetic differences (e.g. gender related) are associated with variations in the vagal tone (Evans et al. 2001) and the acute autonomic responses (e.g. to myocardial infarction) (Huikuri et al. 1996).

### The cholinergic anti-inflammatory pathway and hyperinflammation

**Fig. 1:**
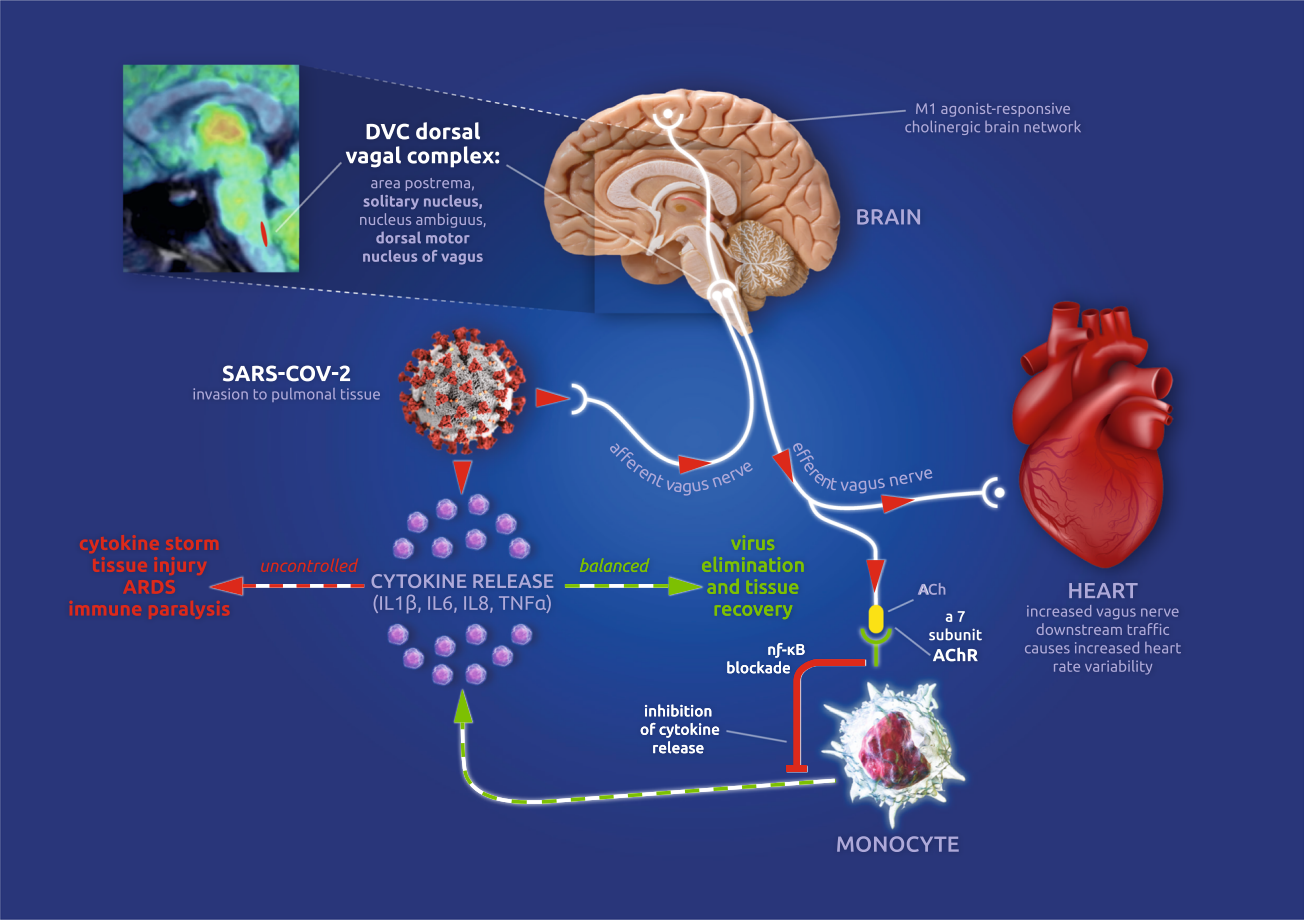
The cholinergic anti-inflammatory pathway (CAP) and its impairment in the case of SARS-2-CoV infection. Cellular virus invasion leads despite local liberation of pro-inflammatory cytokines (i.e. IL 1β, IL 6, IL 8, TNF α) to afferent vagal signaling. In central vagal structures (solitary nucleus (NTS), dorsal motor nucleus of the vagus (DMV)) this signal is after receiving (NTS) and interactional communication to different central instances via a M1 agonist responsive cholinergic brain network transformed into an appropriate efferent vagal impulse (DMV), which is liberating acetylcholine (ACh) at the spot of injury. Thus, cytokine distribution is controlled by somatotopically vagal secreted acetylcholine (ACh) coupling to α7-nicotinergic-acetylcholine receptors (α7AChR´s), this way blocking the cytokine transcriptional factor nuclear factor kappa B (nf-κB). This mechanism is dependent on appropriate parasympathetic (vagal) representation within the autonomic nervous system (ANS). Depressed vagal tone leads to functional impairment of CAP with subsequent excessive cytokine distribution (cytokine storm) followed by tissue injury, pulmonal dysfunction, ARDS and immune paralysis, whereas in the case of ANS balance, controlled cytokine distribution is generating tissue repair and virus elimination

Borovikova (Borovikova et al. 2000) and Tracey (Tracey 2002) first described the cholinergic anti-inflammatory pathway (CAP). CAP controls cytokine release via damage-associated afferent vagal signaling (Borovikova et al. 2000; Tracey 2007; Tracey 2002) onto the dorsal vagal complex (DVC). DVC stands for a group of vagal neurons (area postrema - AP, ambiguous nucleus - NA, solitary nucleus - NTS, dorsal motor nucleus of the vagus - DMV) in the brainstem (Travagli et al. 2006). These central parasympathetic structures are responsible for receiving and processing that vagal afferent signaling (NTS). Muscarinic (M1) agonist network communication to certain neural groups modulates the signal to create a somatotopic efferent vagal impulse (DMV) (Tracey 2002). This impulse leads to acetylcholine (ACh) secretion at the site of injury (Tracey 2007; Tracey 2002). ACh couples to α7 subunit-characterized nicotinic ACh receptors (α7nAChR’s), in turn stopping the action of nuclear factor κB (NF-κB), the transcriptional factor (TF) (Tracey 2007). Thus, it prevents further transcription and downstream release of NF-κB-dependent pro-inflammatory cytokines (Borovikova et al. 2000; Tracey 2007; Tracey 2002). Equilibration between pro- and anti-inflammatory processes is thereby achieved and provides innate immunity without damaging host tissue (Tracey 2007). (Fig. 1). The spleen, as the principal source of pro-inflammatory cytokines, amplifies local cytokine liberation up to systemic cytokine storm and its involvement as main target of the CAP via catecholaminergic splenic nerve fibers is essential for CAP functionality (Huston et al. 2006).

Since controlled cytokine release is imperatively linked to a well-balanced autonomic nervous system (ANS) (Tracey 2002), a controlled pro-inflammatory cytokinergic response to the SARS-CoV-2 invasion appears unlikely in patients with the aforementioned vagus-compromising, pre-existing conditions.

### The coronavirus hijacks NF-κB transcriptional pathway

**Fig. 2:**
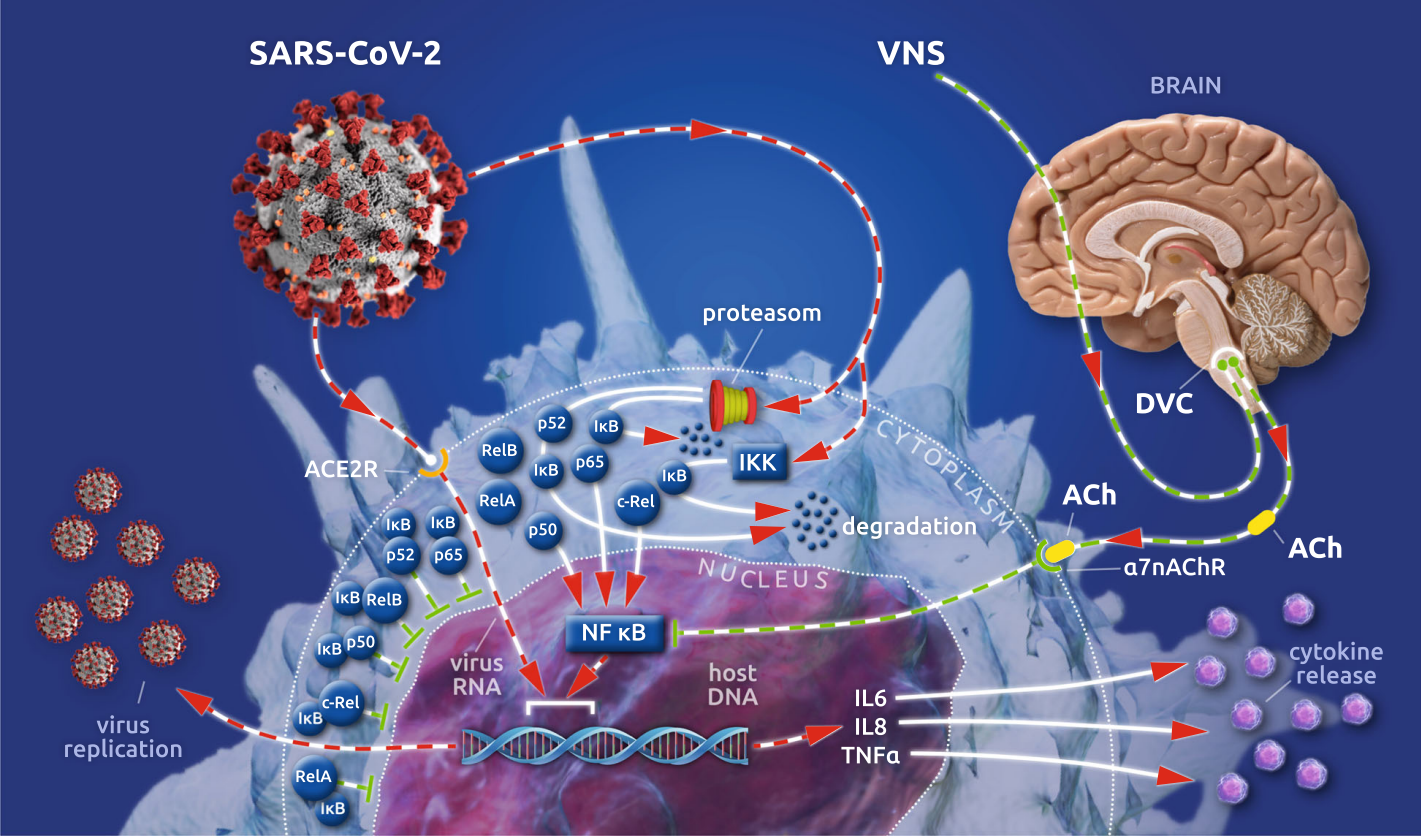
(Key Figure) The cellular virus invasion (SARS-CoV-2) with viral hijacking and amplification of the nuclear factor kappa B (NF-κB) pathway for its replication with additional excessive release of pro-inflammatory cytokines (cytokine storm). In the normal cell cycle, subunits (p65, p50 or p52, c-Rel, RelA or RelB) of the transcriptional factor of numerous pro-inflammatory cytokines (NF-κB) are bound by inhibitor proteins of NF-κB subunits (IκB) in the cytoplasm, which leads to regulation of NF-κB production. After cellular virus invasion (SARS-CoV-2) via the ACE 2 receptor, the virus amplifies the activity of the IκB kinase complex (IKK) catalyzing the proteolytic degradation of IκB by the proteasome. This in turn leaves free NF-κB subunits to translocate unrestricted to the nucleus, where they form NF-κB. NF-κB binds to nuclear DNA in order to enhance virus replication as well as the synthesis and release of pro-inflammatory cytokines (i.e. IL 6, IL 8, TNF α). Vagally secreted acetylcholine (ACh) binds to α7 nicotinic acetylcholine receptors (α7nAchRs) at the cellular surface, blocking the NF-κB action and controlling both, virus replication and release of pro-inflammatory cytokines. Afferent vagal nerve stimulation leads to rebalanced vagal representation in a hypersympathetic, imbalanced autonomic nervous system (ANS), which can be seen in several medical conditions, and during stress in general, but also along the course of critical illness such as severe forms of COVID-19 itself

Coronaviruses use the NF-κB pathway for their replication (Poppe et al. 2017). Under unimpaired conditions cytoplasmatic pre-existing subunits of NF-κB (p65, p50, or p52, c-Rel, RelA or RelB) (see Table 4) are bound to inhibitor proteins (IκB) (see Table 4), preventing their passage through the nuclear membrane (Poppe et al. 2017). They are set free along the NF-κB pathway. This release of the subunits is executed via proteolytical degradation of IκB. When a coronavirus invades the cytoplasm, this process is initiated by viral-activated IκB kinase complex (IKK) (see Table 4) followed by proteasome-derived (see Table 4) proteolytic degradation of IκB. In turn, it brings about the nuclear translocation of the NF-κB subunits to form mature NF-κB heterodimers inducing transcription of NF-κB dependent proteins. Thereby both, NF-κB driven virus replication and cytokine syntheses, are accelerated (Poppe et al. 2017). Since CAP controls NF-κB action by ACh coupled to α7nAChR (Tracey 2007), inadequate vagal representation is the cause of both unrestricted virus replication and uncontrolled cytokine release along the viral-hijacked NF-κB pathway (Poppe et al. 2017). (Fig. 2, Key Figure).

**Table 4:** Proteins regulating cellular clearance - dysregulated by viral invasion

As **subunits of NF-κB** the homodimers of p65, p50 and p52 (non-Rel-dimers) are, in general, repressors of κB transmission. Homodimers with a RelA, RelB or c-Rel-domain (Rel-dimers) and an additional transactivation domain function as activators of NF-κB related transcription after heterodimerization with non-Rel-dimers. Practically heterodimerization between all the homodimers is possible. But only heterodimers composed of non-Rel- and Rel-dimers have transcription activating function.	
Inhibitors, called **IκBs (Inhibitors of κB)**, sequester the NF-κB homodimers in the cytoplasm of unstimulated cells. They do so by using multiple copies of a sequence called ankyrin repeats. By virtue of the ankyrin repeat domains, the IκB proteins mask the nuclear localization signals (NLS) of homodimeric NF- κB proteins. Thus, nuclear translocation of NF-κB dimers is prevented keeping them sequestered in an inactive state in the cytoplasm.	
**Proteasomes** are large (1700 kDa) cytosolic protein complexes which degrade unneeded or damaged proteins by proteolysis which is a key function in cellular homeostasis and survival. To allow proteolytic degradation, the target proteins are tagged with small (8,5 kDa) proteins called ubiquitins. This tagging is catalyzed by enzymes called ubiquitin ligases. In the case of IκBs the viral induced **IκB kinase complex (IKK)** functions as ubiquitin ligase promoting cleavage of IκB- NF-κB-homodimercomplexes thus, allowing nuclear translocation of NF-κB subunits to form NF-κB.

Once the cytokine storm has started causing critical illness, depression of the vagal compartment of the ANS becomes a prognostic factor describing the severity of critical illness (Arbo et al. 2020; Pong et al. 2019). This perpetuates the hyperinflammatory syndrome and makes a therapeutic breakthrough a seemingly futile venture.

## Concluding remarks and future perspectives

Our observations, together with previous investigations, led us to hypothesize, that individuals with unimpaired ANS conditions, respond to SARS-CoV-2 infection with a well-balanced, innate, and adaptive immune response causing mild symptoms of COVID-19. This balancing is conducted via the vagus nerve-driven CAP, limiting NF-κB action in cytokine transcription. In addition, the dynamics of viral replication seem to be inversely correlated with vagal signaling, due to the replications NF-κB dependency. Thus, endogenous parasympathetic regulatory circuits are involved in the control of viral load.

**Table:** 

**Outstanding questions** • Is vagal nerve stimulation capable of stopping critical illness and cytokine storm, or is it just preventive to avoid severe COVID-19 courses? • Can vagal nerve stimulation reduce the incidence of extrapulmonary organ failure? • Which vagus nerve stimulation approach is qualified for the use in viral infections? • Since stimulation parameters are particularly important in vagus nerve stimulation approaches, which parameters are suitable under a virus-driven cytokine storm? • Are there adverse effects of vagal nerve stimulation in the treatment of COVID-19? What role does the initial virus load play? • Are there contraindications for vagal nerve stimulation in the field of infectious hyperinflammatory syndromes?	

We believe that sympatho-vagal balance and vagal nerve stimulation (VNS) should be taken into consideration when evaluating diagnostic and therapeutic approaches to severe courses of COVID-19. This can be achieved diagnostically by measuring heart rate variability (HRV) (see Glossary), which is a widely accepted, noninvasive diagnostic method for the evaluation of autonomic balance (Schwerdtfeger et al. 2020). Moreover, HRV-evaluation is cost-neutral and available for use under study- and clinical conditions (Schwerdtfeger et al. 2020; Gevirtz 2015). Thus, efforts should be made to observe correlation between ANS balance compromising disorders and severity of SARS-CoV-2 infection courses. In numerous disease patterns related to uncontrolled cytokine release, VNS has been established to be part of the therapeutic approach (Beekwilder and Beems 2010; Milev et al. 2016; Koopman et al. 2016; Merrill et al. 2006; Bonaz et al. 2016; Bonaz et al. 2013; Albert et al. 2015; Berry et al. 2013; Critchley et al. 2007; Guarini et al. 2003; Hoshide and Jandial 2018; Neren et al. 2016). Moreover, VNS has been shown to inhibit NF-κB action (Guarini et al. 2003). In a preclinical study, VNS was proven to prevent ARDS development, after scorpion poison (mesobuthus tamulus) had been administered (Akella and Deshpande 2015).

Due to its propensity to bind not only to ACE 2 receptors but most likely to nAChR’s as well, SARS-CoV-2 is hypothesized to be capable inducing a primary or secondary neuroinfection (Changeux et al. 2020; Steardo et al. 2020). These infections differ on one hand symptomatically and prognostically from the courses with primary invasion of the respiratory tract cells and on the other hand suggest the high effectiveness of nicotine as a therapeutic agent competing with SARS-CoV-2 for nAChR’s thus, ameliorating COVID-19 courses (Changeux et al. 2020). The presumed nAChR assisted cell invasion is as scientifically interesting as is the opportunity for the virus to compromise CAP action by blocking of nAChR’s. This so-called `nicotine hypothesis` (Changeux et al. 2020) and our currently introduced hypothesis are to be considered together.

As for peripheral circulating and central administered Angiotensin II (AII), shows direct vagus nerve depressing effects (Vaile et al. 1998) and VNS vice versa could be proven to be cardioprotective to chronic heart failure subjects (Li et al. 2004) both, ANS balance and the renin-angiotensin system (RAAS) show a broad interconnection. This and the described anti-inflammatory potential of ACE antagonistic agents (Di Raimondo et al. 2012) shift the controversy concerning the potentially harmful versus the potentially beneficial effects of angiotensin-converting–enzyme (ACE) inhibitors and angiotensin-receptor blockers (ARBs) clearly into the beneficial column and the preventive withholding of these drugs to prevent severe COVID-19 courses appears to be no longer worth discussing (Speth 2020).

The cardiac proarrhythmic pathognomonic and therapeutic dilemma mentioned above is complicated by the fact that drugs commonly used in severe COVID-19 cases (chloroquine/hydroxychloroquine, lopinavir/ritonavir, protease inhibitors, macrolides, fluoroquinolones) have a QT prolonging potential themselves. Especially the use of the IL 6 monoclonal antibody (tocilizumab), which seemingly reduces mortality in COVID-19, is limited by its hERG-K+ channel blocking and thus pro-arrhythmic characteristics (Lazzerini et al. 2020b).

Therefore, we believe that VNS could be one therapeutic key to avoid or to attenuate severe COVID-19 courses. There are several methods described for VNS (Johnston and Webster 2009). We know direct electrical efferent and afferent VNS. Moreover, several transcutaneous VNS (Baig et al. 2019) approaches are also in use (Johnston and Webster 2009). Recently, Staats et al. (Staats et al. 2020) reported distinct symptom relief (fatigue, low appetite, and coughing bouts such as dyspnea and chest pressure/tightness) in two SARS-CoV-2 positive tested patients under the usage of noninvasive vagal nerve stimulation (nVNS). Bonaz et al. (Bonaz et al. 2020) suggest to target CAP in the treatment of COVID-19 patients using VNS and utilize its proven cytokine controlling, anti-inflammatory potential. Moreover, several pharmacological agents (i.e. CNI-1493 (see Glossary) (Oke and Tracey 2007), nicotine, GTS-21 (see Glossary) (Wang et al. 2020b), or ghrelin (Bansal et al. 2012)) are known to enhance vagal signaling (Johnston and Webster 2009). We should mention, that several authors investigating biofeedback methods to increase the vagal tone, found a respiratory frequency of 0.1 Hz to be the resonance frequency to amplify vagal signaling, measured by increasing HRV (Schwerdtfeger et al. 2020; Gevirtz 2015). Further, they were able to show significant amelioration via biofeedback in illness patterns related to sympatho-vagal imbalance or rather elevated serum cytokine levels (Gevirtz 2015; Gevirtz et al. 1996). Because of their wide availability and negligible costs, biofeedback mechanisms should be explored for their potential in restoring ANS balance and therefore preventing severe COVID-19 courses.

Such an exploration becomes urgent, especially when looking at the COVID-19 avalanche coming towards countries with little potential to manage this catastrophe in a sufficient manner.

Many other viruses (PRSSV (see Glossary) (Wang et al. 2013), RSV (see Glossary) (Masaki et al. 2011), HIV-1C (see Glossary) (Dave et al. 2020; Hiscott et al. 2001), HBV (see Glossary) (Hiscott et al. 2001; Liu et al. 2020), HCV (Hiscott et al. 2001), HIV (Pahl 1999), EBV (see Glossary) (Hiscott et al. 2001)) have been proven to use the described NF-κB pathway for replication. This offers a wide area of therapeutic applications for vagal nerve stimulation.

Even if the described hypotheses reflect just a part of the complex pathophysiological processes in viral infections, they might provide direction guiding input for further basic research to combat COVID-19. Nevertheless, some questions concerning VNS in COVID-19 have got to be answered (see outstanding questions). The question if VNS is able to control the cytokine storm, when critical illness has taken already place or if it is capable more likely to avoid such severe courses of COVID-19, needs to be addressed, as well as the question for the effectiveness of VNS in extrapulmonary disease manifestations. Since different approaches of VNS are described (i.e. afferent or efferent electrical VNS, transcutaneous or bio-feedback VNS), these approaches should be investigated with regard to their differences in terms of effectiveness, security and possible contraindications, or adverse effects in critical ill subjects before any clinical use begins. Nonetheless, concerning the high dynamics of the COVID-19 associated pathologic processes, the question for the stimulative patterns in the case of using electrical (transcutaneous or direct) VNS, seems to be crucially important.

## Data Availability

Not applicable.

## Glossary

ARDS: Acute respiratory distress syndrome (ARDS) describes a respiratory failure characterized by rapid onset of widespread inflammation in the lungs. 1
ASC: Apoptosis-associated speck-like protein containing a CARD, also named PYCARD, is mainly stored in the nucleus of monocytes and macrophages. It acts as a pivotal adaptor protein in activation of the inflammasome. 4
CNI-1493: CNI-1493 is a chemical inducing activation of central vagal neurons. 10
EBV: The Epstein–Barr virus (EBV), a herpesvirus, is one of the most common viruses in humans. 11
GTS-21: GTS-2 (DMXBA) acts as a partial agonist on neural nicotinic acetylcholine receptors. 10
HBV: Hepatitis B virus (HBV), is a DNA virus causing hepatitis B. 11
HCV: The hepatitis C virus causes hepatitis C and some cancers, such as hepatocellular carcinoma. 11
HIV: The human immunodeficiency viruses causes the acquired immunodeficiency syndrome (AIDS). 11
HIV-1C: HIV-1C, a HIV subtype predominant in southern Africa 11, 14
HRV: Heart rate variability measures the physiologically inconsistent gaps between each heartbeat and is used as an index to evaluate the balance of the autonomic nervous system. 10, 11, 13
IL 18: Interleukin-18 is a proinflammatory cytokine produced by many cell types and a factor that induces interferon-γ (IFN-γ) production. 4
IL 1β: interleukin 1β is an important mediator of the inflammatory response, and is involved in a variety of cellular activities, including cell proliferation, differentiation, and apoptosis. 1, 2, 4, 7
IL 6: Interleukin 6 is an important mediator of fever and of the acute phase response and is secreted by macrophages in response to specific microbial molecules, referred to as pathogen-associated molecular patterns (PAMPs) 1, 2, 4, 7, 9
IL 8: Interleukin 8 is a chemokine secreted by macrophages and other cells such as epithelial cells, airway smooth muscle cells, or endothelial cells. IL-8 acts as a chemokine, attracting immune cells to immigrate. 1, 2, 4, 7, 9
NLRP3: The NLRP3 (also known as NALP3 and cryopyrin) inflammasome is the best characterized inflammasome. It comprises the NLR (Nod-like-receptor) protein NLRP3, the adapter ASC and pro-caspase-1. 4
PRR: Pattern recognition receptors detect molecules specific for pathogens. They are expressed by dendritic cells, macrophages, monocytes, neutrophils and epithelial cells 4
PRSSV: Porcine reproductive and respiratory syndrome virus 11
RSV: Respiratory syncytial virus 11
S-protein: The spike protein is part of the viral envelope and interacts with its complement host cell receptor, which is central in determining the tissue tropism, infectivity, and species range of the virus. 3
TNF α: Tumor necrosis factor alpha is a cytokine promoting systemic inflammation and inducing the acute phase reaction. It is produced mainly by activated macrophages, but can be produced by many other cell types. 1, 2, 4, 7, 9
